# Treatment of Nasal Catarrh

**Published:** 1881-12

**Authors:** 


### TREATMENT OF NASAL CATARRH.

Dr. M. M. Griffith, M. D., in the Medical and Surgical Reporter for October 29, says:

Of all the therapeutic agents, I value none more highly than the preparations of petroleum, both locally and constitutional. Having used pills prepared from condensed petroleum, or petroleum mass, very extensively in bronchial and lung diseases, and having been time and again informed by patients that they cured their catarrh, I have used nothing else as a constitutional remedy for several years, and effected a cure in nearly all my cases, unless the catarrh was kept up by hypertrophy of the nasal mucous membrane. The formula I have usually used in making these pills is as follows:

| | | | |
|---|---|---|---|
| ℞. | Petroleum mass., | | ℥j |
| | Pulv. cubebæ, | | |
| | Pulv. ipecac comp., | aa | ℥j |

Make pill mass—four-grain pills.

SIG.—One three or four times a day.

Us[illegible] chlorate of p[illegible]tash in making Dover's powders, instead of sulph[illegible]te. The petroleum and cubebs, by their specific action on the mucous membranes or respiratory tract, heal and soothe; they loosen up the debris which keeps up the irritation. In ozæna their action is palliative; much relief is often obtained; spiculæ of bone are often thrown off and discharged. I formerly used kerosene diluted with milk and used as a spray in an atomimiser; this often gave satisfactory results, but since using the pills I have not found it necessary, only in exceptional cases, to resort to local treatment. I have had but a few patients that found it necessary to use the pills more than two or three months; often cases are cured in as many weeks. The plan is cheap, convenient, and efficacious. It has this to recommend it, that catarrh is often associated with other diseases of the respiratory apparatus, and no more efficacious treatment is found than the preparation above described. It is my sheet anchor in coughs, colds, asthma, bronchitis, and the best palliative in phthisis pulmonalis.

